# The role of cancer-associated fibroblasts in tumorigenesis of gastric cancer

**DOI:** 10.1038/s41419-022-05320-8

**Published:** 2022-10-16

**Authors:** Hui Sun, Xu Wang, Xin Wang, Midie Xu, Weiqi Sheng

**Affiliations:** 1 Department of Pathology, Fudan University Shanghai Cancer Center, 200032 Shanghai, China; 2 Department of Oncology, Shanghai Medical College, Fudan University, 200032 Shanghai, China; 3 Institute of Pathology, Fudan University, 200032 Shanghai, China

**Keywords:** Cancer microenvironment, Long non-coding RNAs, Protein-protein interaction networks, miRNAs, Gastric cancer

## Abstract

Despite advances in anticancer therapy, the prognosis of gastric cancer (GC) remains unsatisfactory. Research in recent years has shown that the malignant behavior of cancer is not only attributable to tumor cells but is partly mediated by the activity of the cancer stroma and controlled by various molecular networks in the tumor microenvironment (TME). Cancer-associated fibroblasts (CAFs) are one of the most abundant mesenchymal cell components of the stroma and extensively participate in the malignant development of GC malignancy. CAFs modulate the biological properties of tumor cells in multiple ways, including the secretion of various bioactive molecules that have effects through paracrine and autocrine signaling, the release of exosomes, and direct interactions, thereby affecting GC initiation and development. However, there is marked heterogeneity in the cellular origins, phenotypes, and functions of CAFs in the TME of GC. Furthermore, variations in factors, such as proteins, microRNAs, and lncRNAs, affect interactions between CAFs and GC cells, although, the potential molecular mechanisms are still poorly understood. In this review, we aim to describe the current knowledge of the cellular features and heterogeneity of CAFs and discuss how these factors are regulated in CAFs, with a focus on how they affect GC biology. This review provides mechanistic insight that could inform therapeutic strategies and improve the prognosis of GC patients.

## Facts

- As one of the main components in the tumor environment (TME), cancer-associated fibroblasts (CAFs) are extensively involved in the progression of malignant tumors, including GC.
- CAFs modulate the biological properties of GC cells in multiple ways, including the secretion of various bioactive molecules, the release of exosomes, and direct interactions.
- CAF heterogeneity may be probably related to the diversity of cellular origin, phenotype, and function in GC CAFs.

## Open questions

- What is the regulatory role of CAFs in the TME of GC?
- Which mechanisms are mediated by CAFs to promote GC malignant progression?
- What is the mechanism by which proteins, miRNAs, and lncRNAs participate in crosstalk interactions between GC cells and CAFs?
- How can CAFs be used to improve the dilemma of GC clinical treatment?
- What is the future direction of CAF-based GC therapy?

## Introduction

Gastric cancer (GC) is one of the most prevalently diagnosed malignancies in the world and the leading cause of cancer-related mortality [1]. Although it has a decreasing incidence globally, the incidence remains relatively high in China [2]. Despite recent progress owing to adjuvant treatment and surgery having been confirmed to make great progress in GC, the prognosis of patients with advanced GC remains unsatisfactory due to the high rate of tumor recurrence and distal metastasis [3]. Thus, there is a pressing need to investigate the molecular mechanisms of GC pathogenesis and progression to increase the treatment response of patients.

Stephen Paget proposed the “Seed and Soil” theory in 1889, in which he postulated that cancer cells (seeds) primarily grow in the proper medium (soil) of select tissues, and this idea has proven true in multiple studies of tumor growth and metastasis [4]. However, the prevailing view of tumorigenesis during the past five decades, which mainly emphasizes the “seed”, is the somatic mutation theory (SMT) [5]. This theory has led cancer researchers to focus on tumor genomics and the design of cancer therapies around the druggable characteristics of cancer epithelia, yet the potential function of the tumor stroma has been ignored. Indeed, it has become definitely clear over the past 20 years that cancer progression is not a cell-autonomous process but rather based on the intriguing interaction between cancer cells and the tumor microenvironment (TME) [6, 7]. Indeed, the TME, mainly composed of the extracellular matrix (ECM), stromal cells, cancer stem cells, cancer cells, immune cells, pericytes, endothelial cells (ECs), and cancer-associated fibroblasts (CAFs), has been widely implicated in tumorigenesis and progression in different types of cancer, including GC [8].

The definition of CAFs is usually applied as an umbrella term to refer to a sophisticated and heterogeneous group of activated stromal cells with functions that differ from those of normal fibroblasts (NFs). CAFs can promote cancer invasion and metastasis by inducing biochemical changes and regulating tumor-related signaling [9]. Nevertheless, a fact related to CAFs that has been ignored by various studies is that CAFs exert a negative influence on malignant tumorigenesis and progression under certain conditions [10]. The high heterogeneous expression patterns of CAF biomarkers reflect their cellular origin, phenotype, and function. In this review, we attempt to discuss the cellular features of CAFs in the TME of GC, with an emphasis on their heterogeneity and functional diversity.

## The heterogeneity of CAFs

### Heterogeneity in CAFs origin

**Fig. 1:**
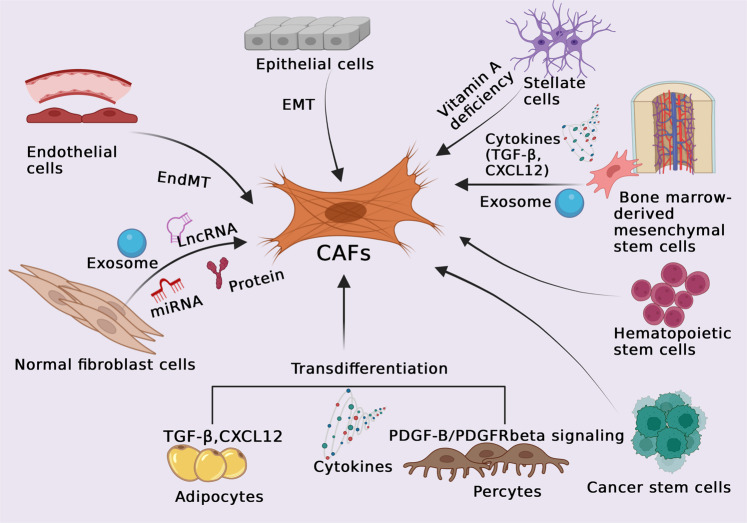
Origin heterogeneity of CAFs. CAFs comprise a complex and heterogeneous group of cells. This heterogeneity might be attributed to the diversity of CAF origins. Numerous cells can be activated and recruited as CAF precursors, such as (1) NFs, (2) epithelial cells (through the epithelial-mesenchymal transition; EMT), (3) endothelial cells (through the endothelial-mesenchymal transition; EndMT), (4) peritumoral adipocytes, (5) pericytes, (6) hematopoietic stem cells, (7) bone-marrow-derived mesenchymal stem cells (BMSCs), and (8) cancer stem cells. It has been indicated that NFs can be educated and further transformed into CAFs through cytokine and chemokine activation in GC. Furthermore, CAFs can also arise from cancer stem cells, hematopoietic stem cells, and BMSCs.

Emerging studies have reported that CAFs comprise a complex and heterogeneous group of cells. This heterogeneity might be attributed to the diversity of CAF origins [11]. There is growing evidence that numerous cells can be activated and recruited as CAF precursors (Fig. 1) [12], such as (1) NFs, (2) epithelial cells (through the epithelial-mesenchymal transition; EMT), (3) endothelial cells (through the endothelial-mesenchymal transition; EndMT), (4) peritumoral adipocytes, (5) pericytes, (6) hematopoietic stem cells, (7) bone-marrow-derived mesenchymal stem cells (BMSCs), and (8) cancer stem cells. It has been indicated that NFs can be educated and further transformed into CAFs through cytokine and chemokine activation in GC [13, 14]. In general, epithelial cells and endothelial cells undergo EndMT or EMT, respectively, and acquire a fibroblastic phenotype in breast cancer [15]. Although adipocyte and pericyte transformation into CAFs is not a common phenomenon in cancers, it has been observed in some human tumors [16, 17]. Furthermore, CAFs can also arise from cancer stem cells, hematopoietic stem cells, and BMSCs [12, 18, 19]. The diverse origins of CAFs explain their heterogeneous features of CAFs, at least to a certain degree.

### Phenotypic heterogeneity of CAFs

The heterogeneity of CAF in phenotype might be due to diverse CAF molecular markers and their precise expression patterns within the specific TMEs. Numerous biomarkers have been considered CAF markers, including but not limited to: alpha-smooth muscle actin (α-SMA), fibroblast activation protein (FAP), fibroblast specific protein 1 (FSP1/S100A4), vimentin, podoplanin (PDPN),platelet-derived growth factor receptors (PDGFRα/β), caveolin-1, meflin, CD10, and GPR77 [20–23] (Table 1). However, none of these biomarkers is specifically expressed in CAFs, and every biomarker represents an independent CAF subset with partially overlapping molecular characteristics. Therefore, none of these markers are able to represent all CAF subsets or distinguish specific CAFs from other kinds of cells, highlighting the heterogeneity in CAF phenotypes.

**Table 1:** Commonly used CAF markers.

Marker	Cell origin	Expression level in CAFs	Biological functions	Effects on GC	Refs.
α-SMA	NFs, pericytes, smooth muscle cells	Upregulated (downregulated in prostate cancer)	Cell contractility, motility, structure, and integrity	Prognostic and postoperative chemotherapy indicator	[24]
FSP1	NFs, epithelial cells, endothelial cells	Upregulated	Cell motility, tissue fibrosis	Not known	[32]
FAP	NFs, quiescent stellate cells, immune cells	Upregulated	ECM remodeling and fibrogenesis	Induce angiogenesis and promote metastasis	[30]
Vimentin	Endothelial cells	Upregulated	Cell motility, structure, and integrity	Induce EMT and migration	[33]
PDGFR	NFs, smooth muscle cells, pericytes	Upregulated	Receptor tyrosine kinase activity	Induce EMT and promote metastasis	[38]
PDPN	Endothelial cells	Upregulated	Cell motility and adhesion	Prognostic indicator	[34]
Caveolin-1	NFs, endothelial cells, adipocytes	Downregulated	Structure component	Prognostic indicator and EMT	[41]
Meflin	undifferentiated mesenchymal stem cells	Downregulated	maintain the undifferentiated state of MSC	Not known	[40]
CD10	Breast MSCs, pre-B lymphocytes	Upregulated	metalloendoprotease	Not known	[43]
GPR77	Polymorphonuclear neutrophils	Upregulated	Complement activation, pro- inflammatory signaling	Not known	[43]

The phenotypic heterogeneity of the CAFs in the TME of GC has also been confirmed in different studies. For instance, α-SMA not only contributes to distinguishing CAFs with a myofibroblastic phenotype in several tumors (a remarkable exception is the downregulation of α-SMA in the prostate cancers matrix) but also acts as a common biomarker of stromal cells, including smooth muscle cells and vascular pericytes [24–26]. Furthermore, CAFs with lower α-SMA expression can promote cell proliferation but inhibit the self-renewal of oral stem-like cancer cells through bone morphogenetic protein 4 (BMP4) [27]. These results demonstrate the heterogeneity of α-SMA^+^CAFs, which has also been recently proven in GC research. Specifically, α-SMA^high^B7-H3^high^ CAFs predict poor prognosis in GC [28]. As a surface marker for CAFs, FAP participates in ECM remodeling and fibrosis through the activity of serine protease, consequently facilitating cancer progression [10]. FAP^+^CAFs may aid in the establishment of an immunosuppressive TME by producing different chemical mediators, such as chemokines and cytokines [29]. Recently, it was reported that FAP^high^CAFs are associated with angiogenesis and metastasis in GC [30]. FSP1^+^CAFs promote tumor metastasis and immune evasion in many tumors, including GC [31, 32]. Vimentin is a biomarker of EMT, regulating tissue structure and motion during cell migration, and it has been implicated in CAF motility in GC [20, 33]. PDPN^+^CAFs have also been reported as biomarkers and may reflect the poor prognosis in GC [34].

Recently, single-cell RNA-seq analyses have shown that CAFs derived from hepatic stellate cells can be divided into myofibroblastic CAFs (myCAFs), inflammatory CAFs (iCAFs) and mesothelial CAFs (mesCAFs) [35]. Specifically, CX- chemokine ligand 12 (CXCL12) functions as a biomarker of iCAFs in GC [36]. Moreover, the expression level of PDGFRα is increased in iCAFs, whereas that of PDGFRβ is increased in myCAFs [37]. Guo et al. found that high expression of PDGFRβ is related to GC progression [38]. Thus, PDGFRβ may serve as a biomarker of myCAFs in GC.

The characteristics of CAF heterogeneity have revealed some underlying antitumor CAF subsets and molecular markers. Mesenchymal stromal cell- and fibroblast-expressing Linx paralog (Meflin) is a recently reported CAF marker that labels cancer-inhibiting CAFs in pancreatic cancer(PC) [39]. Meflin expression is decreased via TGF-β pathway induction, showing that inhibition of Meflin may be related to the phenotype of protumor CAFs [40]. There have also been attempts to focus on the cellular characteristics of CAFs as negative regulators of GC progression under certain circumstances. Caveolin-1 also serves as a CAF marker, and its downregulation enhances EMT in GC cells by targeting E-cadherin [41]. To date, strategies using CAFs as a defensive cellular therapeutic are immature; however, the biomarkers might be beneficial for prognostic analysis.

In addition to the CAF markers mentioned above, several other markers have attracted our interest in other human tumors, including CD10 and GPR77, which have also been highlighted [10, 21, 42, 43]. It has been reported that chemoresistant CD10^+^GPR77^+^CAFs can also promote drug resistance in breast cancer cells [44]. CAF subsets with distinct biomarkers that are coexpressed in cancer cells and exert diverse biological effects continue to be identified in different cancers, including GC. However, because of the organ heterogeneity and specific classification criteria, markers and nomenclature applied in different laboratories, our understanding of CAF subpopulations is extensive but currently unclear. We believe that some combination of biomarkers may constitute a superior tool for identifying heterogeneous populations of CAFs in the future.

### Functional diversity of CAFs

**Fig. 2:**
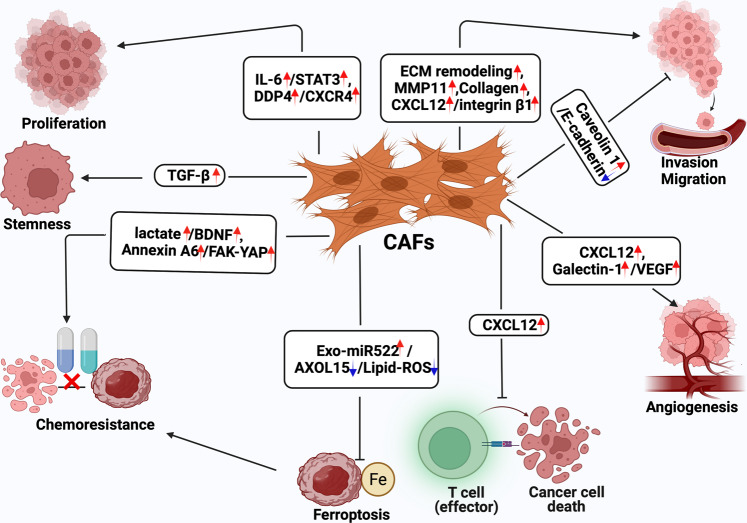
Functional heterogeneity of GC CAFs. CAFs play an essential role in the process of tumorigenesis and cancer progression such as GC cell proliferation, stemness, metabolic changes, and chemoresistance, as well as invasion, migration, EMT, angiogenesis, and immunosuppression in GC by releasing multiple ECM proteins and regulatory molecules.

Given that CAFs differ from NFs, the precise mechanisms by which they function, as well as their impact, remain largely unknown. In general, CAFs play an essential role in the process of tumorigenesis and cancer progression by releasing multiple ECM proteins and regulatory molecules (Fig. 2) [45, 46].

#### CAFs contribute to GC cell proliferation

Unlike NFs, CAFs mediate the development of malignant tumors in benign nontumor epithelial cell lesions. This effect was first noticed in a mouse model of human prostate cancer, in which benign epithelial cells were co-implanted with CAFs [47]. In this model, CAFs caused the cancerous transformation of immortalized benign epithelial cells and drove tumor progression. In agreement with this, co-implantation of tumor cells with CAFs favors carcinogenesis, and tumor proliferation is associated with the co-implantation of tumor cells and NFs in various tumor xenograft models [48]. In GC, this positive effect on tumor cell proliferation of CAFs might be mediated by CAF-released dipeptidyl peptidase-4 (DPP-4) and its receptor, C-X-C chemokine receptor 4 (CXCR4), which is present in tumor cells [49]. CAF-derived IL-6 induces STAT3 activation, which facilitates GC cell proliferation [50].

#### CAFs contribute to GC cell stemness

CAFs have been reported to facilitate the maintenance of breast cancer cell stemness via the CCL2/NOTCH1 pathway [51], and perostin, which is an important component of the ECM derived from fibroblasts, is essential for sustaining breast cancer stemness [52]. Additionally, a recent study also demonstrated that CAFs play an important role during the maintenance of GC stemness [53]. More specifically, a CAF-conditioned medium can stimulate spheroid colony formation and upregulate GC stem cell marker expression, which is inhibited by TGFβ inhibitors, suggesting that CAFs can regulate GC cell stemness via the activity of TGFβ [53].

#### CAFs facilitate invasion, migration, and EMT in GC

Numerous studies have proven that CAFs are able to promote cancer invasion and migration through close interaction with tumor cells, which is also thought to be a feature of CAFs [36]. Through these interactions, soluble factors secreted by CAFs in conditioned media can enhance the invasiveness of cancer under culture conditions. Further study shows that CXCL12 derived from CAFs can promote GC cancer cell invasion by promoting the clustering of integrin β1 on their surface [36]. In addition to inducing the release of cytokines, CAF-mediated TME remodeling boosts tumor invasion and migration. Collagen-rich matrix can promote EMT and the invasion of GC cells [54]. Notably, CAFs may generate gaps in stromal components and the basement membrane that are connected via cell–cell junctions to mediate collective cancer cell migration by MMP-dependent or MMP-independent mechanisms [55, 56]. One study in GC has shown that CAFs can promote GC migration and metastasis in an MMP-dependent manner [57].

#### CAFs control angiogenesis

Angiogenesis is essential for tumorigenesis and progression because the formation of new blood vessels provides nutrition and oxygen for cancer cell progression. CAFs facilitate angiogenesis to maintain the requirements for malignancy proliferation. As mentioned above, CAFs produce CXCL12, which can stimulate neovascularization via the recruitment of bone-marrow-derived endothelial progenitor cells in vivo [48]. CAFs release proangiogenic factors, such as VEGFA, PDGFC, and FGF2, to stimulate or adversely affect angiogenesis in neoplastic tissues [58]. Galectin-1, a 14-kDa carbohydrate-binding protein with an underlying proangiogenic effect, is highly expressed in GC CAFs and can accelerate angiogenesis in GC by promoting VEGFR2 phosphorylation and VEGF expression [46]. Additionally, it has also been shown that CAFs may directly stimulate tumor angiogenesis via paracrine CXCL12 signaling in GC [59].

#### CAFs control immunosuppression

Several studies have shown that CAFs play a vital role in regulating the immune system by releasing cytokines, further resulting in impaired anticancer immune responses. Significantly, macrophage polarization has also been reported in prostate cancer, in which CAFs secrete numerous cytokines, such as IL-6 and CXCL12, and cause tumor-associated macrophages (TAMs) to transition into a tumor-promoting phenotype [60, 61]. In addition, CAFs can produce CXCL12, which suppresses the anti-GC effects of T cells in the TME [62]. Previous studies have also reported that CAF-derived exosomal OIP5-AS1 enhances T-cell tolerance and immune escape by downregulating miR-142-5p and upregulating PDL1 [63]. The ability to modulate blood vessels and immunocytes ultimately underlines the plasticity of CAFs and the probability of targeting CAFs in antitumor therapy.

#### CAFs support GC progression through metabolic changes

Along with cancer cell progression, CAFs provide many nutrients and undergo distinct metabolic reprogramming, which may act as important factors promoting tumor progression [64]. CAF metabolic reprogramming has been observed in several kinds of cancers, including breast cancer, lung cancer, and prostate cancer [65–68]. Exosomes secreted into the microenvironment can modulate cancer cell metabolism [69], and CAF-secreted exosomal miRNAs may participate in cancer cell metabolism [70]. Research on breast cancer suggests that extracellular vesicles can carry miR-105 from cancer cells to CAFs, inducing the metabolic reprogramming that enables CAFs to alter the metabolic environment according to diverse conditions [68]. In addition, metabolic reprogramming may occur in GC CAFs. For example, a recent study confirmed that CAF-derived miR-522 is able to suppress ferroptosis-related metabolism in GC recently [71].

#### CAFs promote GC cell chemoresistance

Chemoresistance remains a very serious challenge for the successful treatment of various types of tumors. Many studies have demonstrated the response of CAFs to antitumor treatments and their functions in chemoresistance [71]. Extracellular vesicles from CAFs containing annexin A6 have been reported to induce FAK-YAP activation and tubular network formation by stabilizing β1 integrin, enhancing cisplatin resistance in GC [72]. In addition, miR-522 derived from CAFs also boosts therapeutic resistance to cisplatin and paclitaxel in GC [71]. Furthermore, CAF-derived BDNF promotes chemoresistance to anlotinib in GC cells via TrkB stimulation; thus, blocking the BDNF/TrkB pathway can induce CAFs to effectively overcome anlotinib resistance [73]. These observations further support the viewpoint that the secretome of CAFs is involved in the regulation of cancer chemoresistance.

### The mechanism of crosstalk between CAFs and GC cells

**Fig. 3:**
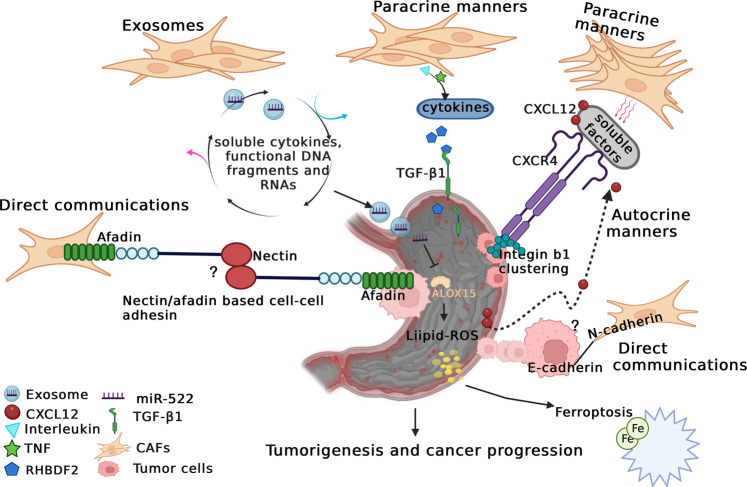
The mechanism of crosstalk interactions between CAFs and tumor cells. The mechanisms by which tumor cells interact with CAFs remain to be elucidated. CAFs and tumor cells may cooperate to invade via diverse communication behaviors, including the chemoattractant gradient produced by soluble cytokines, different secretory patterns, which including paracrine and autocrine signaling, exosomes, and direct communication.

Numerous studies have reported that CAFs play an important role in both malignant transformation and tumor progression through various behaviors [53, 74], but the mechanisms by which tumor cells interact with CAFs remain to be elucidated (Fig. 3). First, CAFs and tumor cells may cooperate to invade via diverse communication behaviors. One of these communication behaviors might be the chemoattractant gradient produced by soluble cytokines to guide cancer cell migration. To a certain extent, the secretory phenotypes of CAFs also show heterogeneity, which is usually presented as different secretory patterns, including paracrine and autocrine signaling. For example, this protumor effect of CAFs can be mediated in both autocrine and paracrine manners by CAF-released CXCL12 and its receptor CXCR4, respectively, in GC [36, 75]. Carcinoma cells themselves occasionally produce CXCL12; more frequently, they foster an environment, including paracrine signaling and cytokines, to stimulate CXCL12 production by stromal cells. High CXCL12 in the TME thus provides paracrine signaling via a feedback loop that mediates integrin b1 clustering at the tumor cell surface, promotes tumor EMT and prevents apoptosis via upregulated CXCR4 on tumor cells. GC cell-derived inflammatory cytokines (such as the interleukin family and TNF) promote RHBDF2 expression in CAFs, mediating transforming growth factor beta 1 (TGF-β1) signaling and enhancing CAF motility in a Smad-independent manner, and further boosting the invasion of GC cells in a paracrine manner [76].

Additionally, exosomes are significant vehicles of genetic information and other material between cells. Exosomes secreted by tumor cells or mesenchymal cells enable the transfer of soluble cytokines, functional DNA fragments, and RNAs, into the mesenchymal cells further to promote their activation. It has been demonstrated that CAF-derived exosome-miR-522 induces acquired chemoresistance in GC cells by targeting ALOX15 and further blocking lipid-ROS accumulation [71]. Exosomes derived from GC cells have also been reported to promote umbilical cord-derived mesenchymal stem cell (UCMSC) migration and differentiation into CAFs via the TGF-β/Smad axis [77].

Finally, CAFs and tumor cells engage in direct communication with each other. Labernadie et al. showed that the mechanical force applied by the heterotypic interactions of E-cadherin/N-cadherin can coordinate invasion between CAFs and tumor cells via two kinds of complex mechanisms: CAFs may enhance the invasiveness of cancer cells by taking them away from the tumor, and cancer cells further boost their spread by directing CAF migration away from the tumor [78]. Nectins, as immunoglobulin-like transmembrane cell adhesion molecules, directly interact with afadin to regulate cell–cell adhesions [79]. Cancer cells are released from locomotion restraints due to the normal contact inhibition provided by surrounding ephrin-expressing noncancer cells. Thus, elevated ephrin levels in prostate cancer cells can promote local invasion [80]. Overall, the contact-mediated signaling pathway, which functions through the Eph/ephrin or nectin/afadin systems, may play a vital role in the crosstalk between cancer cells and CAFs. Despite direct interactions exist between CAFs and cancer cells, the glandular structures of GC tissues sustain the integrity of the basement membrane, which blocks a direct connection with stromal cells [76]. Until recently, almost nothing was known about the direct crosstalk between CAFs and GC cells.

### Molecular communication and networking between GC cells and CAFs

GC cells and CAFs can communicate interactively via various factors and steps [14]. Accumulating evidence has demonstrated that gene mutation plays an essential role in the TME, showing that functional proteins, miRNAs, and lncRNAs selectively expressed in CAFs might be special diagnostic biomarkers and candidate gene targets for GC therapy (Table 2).

**Table 2:** Proteins, miRNAs, and lncRNAs in the effect of CAFs on GC cells.

Moleculars	Expression	Target molecules or pathways	GC cell function change	Refs.
*Proteins*				
Galectin-1	Upregulation in CAFs	integrin β1,VEGF	Promote migration, invasion, and angiogenesis	[46, 99]
Twist1	Upregulation in CAFs	/	Indicate the poorer prognosis	[22]
Caveolin-1	Downregulation in CAFs	HGF,TGF-β, and CXCL12	Induce a CAF phenotype and indicate the poorer prognosis	[23]
RHBDF2	Upregulation in CAFs	TGF-β	Promote invasion	[76]
HGF	Upregulation in CAFs	PI3K/AKT and ERK1/2 signaling	Promote angiogenesis	[82]
Exo-PKM2	Upregulation in CAFs	NF-κB	Inducing abnormal metabolism and inflammation activation	[9]
THBS1	Upregulation in GC cells	/	Promote immunosuppression and chemotherapy Resistance	[83]
CXCL12	Upregulation in CAFs	CXCL12-CXCR4/CXCR7 axis	Promote immunosuppression and invasion	[62]
BDNF	Upregulation in CAFs	Lactate/BDNF/TrkB/ Nrf2	Induce chemotherapy resistance	[73]
Annexin A6	Upregulation in CAF-EV	Integrinβ1- FAK-YAP	Induce chemotherapy resistance	[72]
*MiRNAs*				
Exo-miR-139	Downregulation in CAFs	MMP11	Promotes growth, migration metastasis	[57]
MiR-214	Downregulation in CAFs	FGF9	Promotes migration metastasis and induces EMT	[87]
MiR-141-3p	Downregulation in GC cells	STAT4/wnt/β-catenin	Promotes invasion and migration and transition from NFs to CAFs	[88]
MiR-506	Downregulation in GC cells	ETS1/ miR-506/ECM	Promotes EMT and angiogenesis	[89, 100]
Exo-miR-522	Upregulation in CAFs	USP7/hnRNPA1/miR-522	Suppresses ferroptosis and promotes acquired chemoresistance	[71]
*lncRNAs*				
FLJ22447	Downregulation in CAFs	HIF1A and VEGF	Promotes invasion, migration, and angiogenesis	[59]
NROAD	Upregulation in GC cells	NROAD-miR-496-IL-33 axis	Promote proliferation, migration, invasion, and EMT	[92]

#### Proteins

GC CAFs highly express twist1, and high twist1 expression is associated with poor clinical outcomes; in addition, twist1, a novel CAF marker, can be used to evaluate the prognosis of patients with GC and also serve as a molecular target for GC [14]. In contrast, low expression of caveolin-1, a tumor-suppressor gene, in CAFs predicts adverse outcomes in GC, showing that caveolin-1 in CAFs is probably a useful negative prognostic marker [81]. CAF-derived HGF can promote angiogenesis, vasculogenic mimicry, and mosaic vessel formation through the PI3K/AKT and ERK1/2 pathways in GC [82]. Interaction between GC CAFs and proteins also participates in metabolic responses. Gu et al. reported that PKM2 produced by GC exosomes can promote persistent activation of the NF-κB signaling pathway in CAFs, therefore further contributing to metabolic changes and inflammatory reactions [9]. Moreover, functional proteins play an important role in the regulation of chemoresistance. High expression of THBS1 has been related positively to worse prognosis and immunosuppression, but negatively related to oxaliplatin sensitivity in GC [83]. Blocking the function of these protein-encoding genes associated with CAFs may be used as an alternative treatment for GC therapy resistance in the future.

#### miRNAs

MicroRNAs (miRNAs) are small endogenous RNAs that induce posttranscriptional gene silencing and have been extensively reported to participate in different physiological and pathological processes [45]. Aberrantly expressed miRNAs exert pivotal effects by affecting the level of mRNAs via various signaling pathways, thus further influencing cancer progression [84]. In addition to intracellular regulation, miRNAs can also affect the level of target genes to influence various biological manners of malignancies via intercellular communication [85].

Increasing evidence suggests that CAF-derived miRNAs in the TME regulate different processes of GC cells, including tumorigenesis, development, EMT, and metastasis [46, 86]. Downregulation of CAF-derived miR-214 and exosomal miR-139 enhances the migration and invasion capacities of GC cells in diverse manners [57, 87]. It has also been demonstrated that CAF-derived exosomal miRNAs induce acquired chemoresistance in GC cells by modulating cancer cell metabolism. Cisplatin and paclitaxel have been clearly shown to increase CAF-derived miR-522 secretion by activating the USP7/hnRNPA1 signaling, resulting in lipoxygenase 15 (ALOX15) inhibition and decreasing lipid-ROS accumulation in tumor cells, and finally leading to reduced chemosensitivity [71]. Moreover, CAFs can restrain the anticancer effects of T cells in the GC TME [86]. CAF-derived exosomal OIP5-AS1 boosts T-cell tolerance and immune escape by downregulating miR-142-5p and upregulating PDL1 in lung cancer [63]. However, studies about whether CAF-derived miRNAs regulate immune suppression in GC cells remain limited. Zhou et al. reported that miR-141 suppresses GC cell migration and invasion, as well as the transformation of NFs into CAFs, by targeting the STAT4/Wnt/β-catenin axis [88]. MiR-506 suppresses GC angiogenesis and correlates with downregulated levels of ETS1, which induces tumor vascularization [89].

#### LncRNAs

In general, the capacity of long noncoding RNAs (lncRNAs) to extensively interact with various biomolecules is of great importance in cancer progression [90], and a recent series of experiments has highlighted the roles of lncRNAs in the TME [91]. Furthermore, cancer cells and CAFs can interact with each other more directly via lncRNAs. For example, Huang et al. found that a lncRNA activated by DNA damage (NORAD) was upregulated in GC cells, aggravating the malignant behaviors [92]. Further research found that NORAD increases the pro-GC function of CAFs by targeting miR-496 and upregulating IL-33, offering new insights into the TME of GC cells and CAFs [92]. CAF-derived lncRNA-CAF, also known as FLJ22447, is located in the vicinity of the hypoxia-inducible factor (HIF)-1α gene [59], which plays a vital role in the hypoxic TME and regulates the invasion and migration of cancer cells [93]. Interestingly, low expression of FLJ22447 has been linked to HIF-1α expression in patients with GC [59]. Therefore, FLJ22447 might play an essential role in the interaction between GC cells and CAFs. Although the research on lncRNAs in the TME has just begun, important achievements in our understanding of the effects of lncRNAs on CAFs in the future are likely. Recently, with the rapid development of high-throughput sequencing technologies, lncRNAs have been found to be involved in communication between cancer cells and CAFs [94].

## Conclusions and future perspectives

This review summarizes the features of GC CAFs primarily on the basis of CAF biomarkers, epigenetic alterations, communication molecules, and mechanisms correlating with procancer or anticancer effects, with a special focus on the phenotypical plasticity and functional heterogeneity of CAFs. The problem of CAF heterogeneity highlights other related issues, including whether a single subpopulation of CAFs can simultaneously play diverse roles or whether there are subtypes of CAFs and switching between different functional statuses occurs. Accumulating studies indicate that there is a certain degree of specialization among CAFs [44]. Overall, a consensus regarding CAF subgroups and nomenclature will be essential. To address such issues, some cooperation of CAF markers may be a superior tool to identify the complex and heterogeneous populations of CAFs in the future. Relatedly, the development of multiplex immunofluorescence techniques, which enable the simultaneous analysis of various CAF markers and the identification of more quantitative strategies regarding the relative levels of biomarker expression, may contribute to the reproducible evaluation of CAF subpopulations.

GC also shows inter- and intratumor heterogeneity. Investigators from The Cancer Genome Atlas (TCGA) have uncovered 4 subtypes of GC and characterized their genetic traits by analyzing the existing data of MSI, DNA methylation, Epstein-Barr virus (EBV) status, mutation profiles, and somatic copy-number alterations [95]. It is interesting that the EBV-positive group exhibits PIK3CA mutations, DNA hypermethylation, and amplification of JAK2, PDL1, and PDL2 [95]. Given that PIK3CA signaling and PDL1 are closely related to the activity of CAFs [82, 96], curative strategies with CAFs as targets will likely be preferred for this subgroup of GC patients. Various therapeutic strategies targeting CAFs or their functional mediators have been identified. For instance, several anticancer drugs have already been tested in humans, including smoothened inhibitors(IPI-926) and histone deacetylase (HDAC) inhibitors, which may also target CAFs or their precursors [97, 98]. In general, previous anti-CAF treatment strategies have been studied in terms of understanding the procancer effects of CAFs. Therefore, studies in this field face many difficulties due to the protumorigenic and antitumorigenic effects of CAFs [10]. Future studies are warranted to further clarify CAF heterogeneity, and an understanding of its genetic characteristics will provide valuable insight into the carcinogenic mechanism of GC and might pave the way for the study of new treatment strategies for GC.

Analysis of CAF numbers and types may also be included in clinical studies that are not mainly focused on stromal fibroblast biology, for example, immuno-oncology biomarkers in cancer clinical trials. This will contribute to a more comprehensive understanding of the association between CAFs and therapy responses and emphasize new fields in which CAF-targeted drugs combined with available therapies might yield greater benefits. With the knowledge gained from such experiments, we believe that the CAF-targeted strategy will earn a place in the oncologist’s toolkit in the future.

## Data Availability

The material supporting the conclusion of this review has been included within the article.
